# LONELINESS SHAPES THE RELATIONSHIP BETWEEN ICT USE AND PSYCHOLOGICAL ADJUSTMENT AMONG OLDER ADULTS

**DOI:** 10.1093/geroni/igz038.1950

**Published:** 2019-11-08

**Authors:** Yang Fang, Anson Chau, Helene H Fung, Jean Woo

**Affiliations:** 1 Nanyang Technological University, Singapore, Singapore; 2 Chinese University of Hong Kong, Hong Kong, Hong Kong; 3 The Chinese University of Hong Kong, New Territories, Hong Kong

## Abstract

Background: Given findings that generally support the benefits of information and communication technology (ICT) for older adults’ psychosocial adjustment, one might surmise that lonely older adults, who have a stronger need for psychological support, would reap more psychosocial benefits from ICT use. However, scant research has examined this view, much less the likelihood that ICT use might worsen the psychological well-being of lonely older adults, as has been shown to be the case in younger adults. Objective: To examine whether the association between ICT use and psychological adjustment (i.e., psychological distress and sense of community [SOC]) among older adults depends on their loneliness levels. Methods: A representative sample of 738 Hong Kong SAR Chinese older adults aged 60 years or older (56% female) was interviewed in 2017 on loneliness, frequency of ICT use (i.e., Internet and smart devices), psychological distress (6-item Kessler scale; K6), and SOC. Results: Regression analyses showed that loneliness significantly moderated the relationship between ICT use frequency and psychological adjustment (psychological distress and SOC); more frequent ICT use was associated with more psychological distress and less SOC, with higher levels of loneliness. Conclusion: These findings suggest that ICT use may be a mixed blessing for older adults, i.e., using more ICT might predict worse psychological adjustment among lonelier older adults. Efforts that promote ICT use among older adults should take their loneliness levels into account.

